# Cranial electrostimulation improves slow wave sleep in collegiate population: a polysomnographic study

**DOI:** 10.5935/1984-0063.20220029

**Published:** 2022

**Authors:** Anam Aseem, Neera Chaudhry, Mohammed Ejaz Hussain

**Affiliations:** 1 Jamia Millia Islamia, Centre for Physiotherapy and Rehabilitation Sciences - New Delhi - Delhi - India.; 2 Vardhman Mahavir Medical College and Safdarjung Hospital, Department of Neurology - New Delhi - Delhi - India.; 3 Shree Guru Gobind Singh Tricentenary University, Faculty of Allied Health Sciences - Gurugram - Haryana - India.

**Keywords:** Sleep, Polysomnography, Students, Slow Wave Sleep

## Abstract

**Objective:**

Sleep disturbance is quite prevalent among students, which leads to deleterious consequences on health. Cranial electrostimulation (CES) has been speculated to entrain cortical slow waves; therefore, we investigated the efficacy of cranial electrostimulation to improve slow wave sleep in collegiates.

**Methods:**

Twenty-eight students with Pittsburgh sleep quality index (PSQI) score >5 were randomly assigned into two groups: CES and control. Participants in CES group completed 60 minutes of CES intervention for 12 weeks with 100 µA microcurrent and 0.5 Hz frequency parameters during night. Pre- and post-intervention measures were taken for sleep architecture using over-night polysomnography (PSG) and sleep quality using PSQI. Participants were instructed to report to the laboratory at 10:00 p.m. and PSG was performed with electroencephalograms (EEG), chin electromyography (EMG) and bilateral electrooculogram (EOG) in place. Sleep stages were scored manually in accordance with the new AASM guidelines. PSG variables reported in the present study are sleep latency (SL), total sleep time (TST), percentage of N1, N2, N3, NREM (non-rapid eye movement), REM (rapid eye movement) and sleep efficiency (SE%).

**Results:**

After ascertaining the comparability of demographic and sleep variables at baseline for both the groups, a 2X2 mixed model ANOVA was employed. Significant between-group differences were obtained for N1% and N3% such that N1% decreased and N3% increased post CES. However, other PSG variables, along with PSQI score did not demonstrate statistically significant between-group difference.

**Discussion:**

The present study demonstrated that 12-weeks of CES improved N3% and reduced N1%. Future researches should be undertaken to build upon the findings of present study.

## INTRODUCTION

Disturbance in sleep is an unavoidable health related problem emerging in modern society^1^. Changing lifestyle, work profile, food habits, leisure activities, and different life stresses influence sleep patterns and result in sleep related abnormalities^2^. Despite the strong consensus that sufficient and sound sleep is pivotal in maintaining health, these behaviours are often deprioritized within the typical contemporary lifestyle^3^. Worldwide surveys claim that disturbance in sleep is predominant across various age groups and is considered to be a health epidemic that is often unrecognized, overlooked, under-reported, and that has rather high economic load on the society^4,5^. The prevalence of sleep disturbance is about 30-35% in the general population, which emphasizes the global dimension of this emerging silent pandemic^6^.

The term ‘*sleep disturbance’ is* described as a sub-clinical sleep problem which is perceived subjectively, as an experience of decline from a previously occurring sleep, accompanied by an evidence of impairment in objective sleep assessments, but do not necessarily meet the criteria for a clinical diagnosis^7^. It encompasses disorders of initiating and maintaining sleep, disorders of sleep-wake schedule, and dysfunctions associated with sleep stages^8^. Disturbance in sleep not only impairs quality of life but also pose several health-related consequences^9^. Sleepiness and irregular sleep schedules have many unintended and multifaceted short- and long-term health consequences. Chronic sleep disturbance is related to increase odds of developing hypertension^10^, cardiovascular disease^11^, obesity^12^, metabolic syndrome^13^, diabetes mellitus^14^, and an overall reduced quality of life^15^, whereas acute disturbance in sleep is associated with increased sympathetic outflow^16^, irregularity of autonomic nervous system^17^, and dysregulation of hypothalamic-pituitary-adrenal axis (HPA axis) leading to increased stress responsivity^18^, changes in circadian rhythms, inflammatory responses, immunological dysfunction^19^, emotional distress, mood disorders, and cognitive performance deficits^20^.

Disturbance in sleep has been considered as “an unmet public health problem” and its management and treatment are rarely addressed by medical professionals, despite the large toll it takes on society^9^. There are no formal treatment guidelines in primary or specialty care for the complaints of sleep disturbance^21^. The most common remedy to combat this issue is to sleep longer, catching up sleep on weekends, and to have a better understanding of proper sleep hygiene^22^. Whereas, in situations in which extended work hours are unavoidable, wake-promoting medications/substances such as caffeine^23^, modafinil^24^, and sympathomimetic medications are advised. Since pharmacological drugs are associated with side effects, researchers suggest that there is a need to develop a non-pharmacological intervention to combat this alarming issue of sleep disturbance^25^.

Cranial electrical stimulation (CES) is a non-pharmacological, non-invasive, Food and Drug Administration (FDA) approved method of applying low-intensity electrical current to the brain^26^. The use of CES dates back to 1960s, with a plethora of researches being done to prove its effectiveness in managing various psychophysiological conditions^27-29^. But later, the enthusiasm went down due to paucity of quality researches with optimal controls and randomization procedures to provide us with high level conclusive evidence regarding the utilization of CES to improve sleep. However, there is a revival of this technique nowadays, due to increasing statistics showing sleep irregularities in modern society. In essence, a recent report from systematic review^30^ identified 23 studies, which addressed modulation in sleep with CES in healthy and diseased individuals. Findings illustrated that CES has positive effects on sleep, however, due to heterogeneity in the participants and outcome measures, authors suggested to interpret the results with caution. Considering the overwhelmingly alarming magnitude of sub-clinical sleep disturbances is today’s era, here we evaluated the efficacy of CES to improve sleep using gold standard objective assessment technique, i.e., polysomnography and a validated subjective sleep quality assessment questionnaire, i.e., Pittsburgh sleep quality index (PSQI).

## MATERIAL AND METHODS

### Ethics

The present study was approved by Institutional Ethics Committee (EIC), Jamia Millia Islamia. Research guidelines provided by Helsinki’s declaration, 1964 and its later amendments were followed to implement all the procedures in the study.

### Sample

A sample of 28 male university students who scored >5 on PSQI after screening, were recruited for the present study. PSQI gives information on sleep and disturbance during the previous month. The scale contains 7 subscales including sleep duration, sleep disturbance, sleep latency, daytime dysfunction due to sleepiness, sleep efficiency, overall sleep quality, and sleep medication use, each of which is scored equally between 0 and 3. Individual scores in these 7 domains are summed up to obtain a global score, which ranges from 0 to 21, with >5 as cutoff^31^. All the recruited participants reported being free from previous neurological and/or psychiatric disorders. Participants were excluded if they reported use of alcohol and/or other drug abuse, centrally active medications and/or if they were on sleeping pills.

### Procedure

The study was conducted in sleep and cognition laboratory, Centre for Physiotherapy and Rehabilitation Sciences, Jamia Millia Islamia. Prior to assessment, all the participants were given an information sheet explaining the purpose of study, methodology and their rights as research participants and a written consent was obtained from them before the commencement of study. Initially, participants were asked to report to the sleep and cognition laboratory for 2 consecutive nights around 10:00p.m., at their regular bedtime. They were instructed to avoid caffeine intake after 7p.m. in the evening on the day of sleep study. On night 1, after assessing general demographic (such as age, height, weight, and body mass index), participants were given a familiarization session with all the polysomnography electrodes in place, however no data was recorded. On night 2, complete nocturnal polysomnography recording was performed. After baseline assessment, all the participants were assigned into either of the two groups (CES group, n=14 and control group, n=14) by computer-generated block randomization. After 12 weeks of study duration, both the groups were assessed for sleep architecture using over-night PSG and sleep quality using PSQI.

### Over-night polysomnography

Digital recordings for PSG was performed on RMS polysomnographic system (RMS-Quest 32:51 Polysomnograph-Recorders & Medicare System, Chandigarh, India), which included electroencephalograms (EEG), chin electromyography (EMG), and bilateral electrooculogram (EOG). Before placing the electrodes, the scalp was gently cleaned with isopropyl alcohol and NūPrepTM skin prepping gel (Weaver and Company, U.S.) and Ten20TM conductive EEG paste was applied to different locations on the scalp for electrode placement. Ag-AgCl disc electrodes were secured with a micro pore tape on various recording sites. The standard 10-20 electrode placement system was utilized for EEG recording (F3-M1, C3-M1, P3-M1 and O1-M1 for the left side of the head and F4-M2, C4-M2, P4-M2, and O2-M2 for the right side). EOG was recorded using 2 standard electrodes lateral to each eye, one above and lateral to left eye, and one below and lateral to right eye. For chin EMG, two electrodes were used on both right and left masseter muscle. Sleep stages were scored manually by two different raters in accordance with the new AASM rules for technical performance and scoring of sleep. Both the scorers worked independently and any conflicts were resolved through mutual consensus.

The polysomnography variables reported in the study are sleep latency in minutes- SL (m), total sleep time in minutes-TST (m), percentage of N1 sleep (N1%), percentage of N2 sleep (N2%), percentage of N3 sleep (N3%), percentage of NREM (NREM%), percentage of REM (REM%), and sleep efficiency (SE %)^32,33^.

### Intervention

Participants allocated to both the groups (CES and control) were taught basic sleep hygiene (SH) techniques at the beginning of study.

In addition to SH, participants in the CES group were also administered microcurrent cranial electrotherapy stimulator (CES Ultra, U.S.) for 60 minutes at night, during the initial sleep cycle for 12 weeks, 3 times/week. Participants in the CES group arrived the lab at around 10p.m., after having dinner. After preparation for PSG, they subsequently went to bed, and polysomnographic recordings were started. CES began after the subject entered sleeping state (i.e., after on-line scoring confirmed the presence of N1 sleep for 30 seconds continuously). The micro-current generator was a portable handheld device that was programmed to provide an AC characterized by a modified square wave format, with pulse duration of 2 milliseconds (20% duty cycle). Based on the CES Ultra manual, the current and frequency were set at 100µA and 0.5Hz, respectively. Clip electrodes were attached to both earlobes to deliver micro-current. This level of current intensity was significantly below the human’s threshold of sensation. After the end of the first sleep cycle (as confirmed via on-line scoring), CES was stopped using a knob which could be operated without disturbing the participant.

Participants in this group were instructed to report immediately if they perceived any form of abnormal sensation, headache, nausea, body ache or any other side effect.

Participants in the control group did not undergo any intervention other than SH techniques, which was taught at the start of study. Assessment of criterion measures were taken at baseline and after the completion of study duration (12 weeks).

### Statistical analysis

We utilized Statistical Package for Social Science version 21.0 (SPSS INC., Chicago, IL, U.S.) for data management and analysis. Normality of the outcome variables was assessed by Shapiro-Wilk test and histogram method. Demographic characteristics (age, height, weight, and BMI) were compared between the 2 groups using independent t-test. Polysomnography parameters, along with PSQI score were also compared between the groups at baseline using independent t-test. A 2X2 mixed model ANOVA was employed to examine the effect of CES on the outcome variables and main effects of group (between group differences between CES versus control), time (within group differences between baseline and 12 weeks), and timeXgroup interaction were obtained. A *p*-value of <0.05 was considered significant for all the analysis. Effect sizes are mentioned as partial eta squared (ηp^2^) for variables wherever statistical significance was obtained.

## RESULTS

Table 1 represents the comparison of demographic characteristics between the participants of the groups (CES versus control). Demographic characteristics such as age (*p=*0.31), height (*p=*0.17), weight (*p=*0.37), and BMI (*p=*0.93) were comparable between the groups at baseline as assessed by independent t-test (Table 1).

**Table 1:** Demographic data comparison between two groups (CES and control) using independent t-test. Data are presented as ‘mean (SD)’.

Variables	Study Population	CES	Control	p-value
Age (years)	21.96 (3.86)	22.71 (3.31)	21.21 (4.33)	0.31
Height (cm)	165.57 (6.54)	163.85 (8.16)	167.28 (3.98)	0.17
Weight (kg)	71.53 (7.70)	70.21 (6.67)	72.85 (8.67)	0.37
BMI (kg/m^2^)	25.92 (2.21)	25.89 (2.09)	25.96 (2.41)	0.93

**Notes:** CES = Cranial electrostimulation; cm = Centimetres; kg = Kilograms; BMI = Body mass index.

Polysomnography parameters such as SL (*p=*0.99), TST (*p=*0.18), N1% (*p=*0.49), N2% (*p=*0.57), N3% (*p=*0.30), NREM% (*p=*0.50), REM% (*p=*0.50), and SE% (*p=*0.55), along with PSQI score (*p=*0.32) were comparable when assessed at baseline for between group comparison using independent t-test (Table 2).

**Table 2:** Comparison of polysomnography variables between the two groups (CES and control) at baseline using independent t-test. Data are presented as ‘mean (SD)’.

Variables	Study Population	CES	Control	p-value
SL (m)	79.07 (26.51)	79.00 (18.54)	79.14 (33.41)	0.99
TST (m)	357.32 (44.09)	346.14 (47.90)	368.50 (38.39)	0.18
N1 (%)	20.25 (8.38)	19.14 (8.02)	21.35 (8.88)	0.49
N2 (%)	46.14 (4.56)	45.64 (4.68)	46.64 (4.56)	0.57
N3 (%)	16.00 (5.74)	17.14 (5.94)	14.85 (5.50)	0.30
NREM (%)	82.39 (3.60)	81.92 (2.99)	82.85 (4.18)	0.50
REM (%)	17.60 (3.60)	18.07 (2.99)	17.14 (4.18)	0.50
SE (%)	81.50 (5.01)	80.93 (3.14)	82.07 (6.45)	0.55
PSQI score	9.61 (1.70)	9.29 (1.77)	9.93 (1.63)	0.32

**Notes:** CES = Cranial electrostimulation; SL (m) = Sleep latency in minutes; TST (m) = Total sleep time in minutes; N1% = Percentage of N1 sleep; N2% = Percentage of N2 sleep; N3% = Percentage of N3 sleep; NREM% = Percentage of non-rapid eye movement sleep; REM% = Percentage of rapid eye movement; SE% = Percentage of sleep efficiency; PSQI = Pittsburgh sleep quality index.

Note: one participant from CES group and 2 from control group dropped-out from the present study. However, in accordance with the intention to treat analysis, all the subjects who were randomized to the study and received at least one session of training were included in the final analysis. The baseline values of the participants lost to follow-up were carried forward to replace their missing values at subsequent assessment.

**Figure 1:**
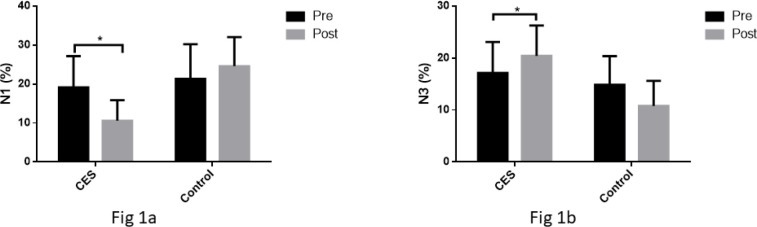
A. Graph demonstrating significant decrease in N1% post 12-week CES intervention with 2X2 mixed model ANOVA statistics; B. Graph demonstrating significant increase in N3% post 12-week CES intervention with 2X2 mixed model ANOVA statistics (*symbolizes significant difference).

Following 12 weeks, 2X2 mixed model ANOVA (Table 3) yielded statistically significant time effect for SL (*p<*0.01; ηp^2^=0.46), TST (*p<*0.01; ηp^2^=0.34), NREM (*p<*0.01; ηp^2^=0.38), REM (*p<*0.01; ηp^2^=0.40), and SE (*p<*0.01; ηp^2^=0.54). However, N1% (*p=*0.06), N2% (*p=*0.22), and N3% (*p=*0.64) demonstrated a non-significant change in time effect post 12 weeks. Moreover, statistically significant interaction effects were observed for SL (*p=*0.02; ηp^2^=0.17), TST (*p<*0.01; ηp^2^=0.33), N1% (*p<*0.01; ηp^2^=0.55), N3% (*p<*0.01; ηp^2^=0.42), REM (*p=*0.05; ηp^2^=0.13), and SE (*p=*0.006; ηp^2^=0.25) after 12 weeks whereas N2% (*p=*0.14) and NREM (*p=*0.06) did not show a significant difference. Interestingly, N1% (*p=*0.05; ηp^2^=0.26) and N3% (*p<*0.01; ηp^2^=0.27) yielded statistically significant effect of group post 12 weeks (Figures 1A and 1B). N1% reduced from 19.14±8.02 to 10.57±5.28 (Figure 1A) and N3% increased from 17.14±5.94 to 20.42±5.84 (Figure 1B) as a result of 12 weeks of CES intervention. Surprisingly, SL (*p=*0.09), TST (*p=*0.46), N2% (*p=*0.87), NREM (*p=*0.09), REM (*p=*0.08), and SE (*p=*0.06) did not demonstrate any statistical significance for main effect of group after 12 weeks of study duration. On the other hand, PSQI score demonstrated a significant difference time (*p*<0.01*; ηp^2^=0.75) and interaction effect (*p=*0.01*; ηp^2^=0.22), however statistically significant difference was not obtained for the main effect of group (*p=*0.73).

**Table 3:** Results of 2X2 mixed model ANOVA demonstrating interaction effect and main effects of group and time for both the groups (CES and control) at baseline and after 12 weeks. ‘*’ indicates significant difference. Data are presented as ‘mean (SD)’.

Variables	CES		Control		Time (p)	Group (p)	TimeXGroup (p)
	Baseline	12^th^ week	Baseline	12^th^ week			
SL (m)	79.00 (18.54)	37.71 (17.48)	79.14 (33.41)	65.42 (30.95)	<0.01*	0.09	0.02*
TST (m)	346.14 (47.90)	410.14 (39.17)	368.50 (38.39)	368.85 (37.92)	0.01*	0.46	<0.01*
N1 (%)	19.14 (8.02)	10.57 (5.28)	21.35 (8.88)	24.57 (7.48)	0.06	0.05*	<0.01*
N2 (%)	45.64 (4.68)	47.85 (3.34)	46.64 (4.56)	46.42 (4.12)	0.22	0.87	0.14
N3 (%)	17.14 (5.94)	20.42 (5.84)	14.85 (5.50)	10.78 (4.83)	0.64	<0.01*	0.01*
NREM (%)	81.92 (2.99)	82.85 (4.18)	82.85 (4.18)	81.78 (3.44)	<0.01*	0.09	0.06
REM (%)	18.07 (2.99)	21.14 (1.46)	17.14 (4.18)	18.21 (3.44)	<0.01*	0.08	0.05*
SE (%)	80.93 (3.14)	91.35 (3.47)	82.07 (6.45)	85.28 (4.82)	0.01	0.06	0.006*
PSQI score	9.29 (1.77)	7.00 (1.71)	9.93 (1.63)	8.71 (1.85)	<0.01*	0.73	0.01*

**Notes:** CES = Cranial electrostimulation; SL (m) = Sleep latency in minutes; TST (m) = Total sleep time in minutes; N1% = Percentage of N1 sleep; N2% = Percentage of N2 sleep; N3% = Percentage of N3 sleep; NREM% = Percentage of non-rapid eye movement sleep; REM% = Percentage of rapid eye movement; SE% = Percentage of sleep efficiency; PSQI: Pittsburgh sleep quality index.

To summarize, inter-group statistically significant differences were demonstrated by N1% and N3% sleep, whereas SL, TST, NREM%, REM%, and PSQI score showed intra-group significant differences. Additionally, significant interaction effect was obtained for SL, TST, N1%, N3%, REM%, SE%, and PSQI score.

Another encouraging finding from the present study is that when asked about side effects, none of the participant reported any form of aches or nausea while using CES.

## DISCUSSION

The present study aimed at exploring the effect of CES on sleep using various polysomnographic parameters. The main findings include reduction in N1% and improvement in N3% with 12 weeks CES intervention. Moreover, this study also demonstrated that CES is a safe method as no participant reported any side effect. Additionally, only one subject from the CES group withdrew from the study supporting the fact that CES is well tolerated and has high adherence rate.

The evidence from previous studies demonstrated mixed results regarding the efficacy of CES in sleep problems. A recent RCT^30^ showed that stimulation with 60 minutes of CES for 3 weeks improved sleep quality in 60 fibromyalgia patients with sleep dysfunction as measured by self-rating questionnaire. Another study^34^ assessed sleep onset latency, nocturnal arousals, sleep efficiency, sleep duration, and awakening time in 21 insomniacs as a result of drug abstinence syndrome using subjective questionnaires. Findings showed that CES training improved sleep duration as compared to control group. Similarly, a recent pilot study^35^ assessed sleep latency, total sleep time, and number of awakenings using sleep log in insomnia patients post CES intervention and their results demonstrated that total time spent in sleep improved in CES group as compared to sham. Noteworthy is that, existing literature has utilized subjective questionnaires to assess sleep. However, a recently published trial^36^ utilized PSG and measured sleep efficiency, sleep latency, time spent in different sleep stages and REM latency after CES intervention. The results showed no change in sleep parameters with CES in non-clinical healthy female population which is contrary to our findings.

Increase in N3% in the present trial supports the speculation that CES entrains slow waves in the brain. This is in agreement with the findings of an earlier study^37^ wherein 27 dementia patients with irregular sleep wake pattern were assessed with nocturnal EEG and their results showed an increase in alpha rhythm along with improvement in sleep wake behaviour post CES administration. Similarly, Kennerly et al. (2004)^38^ assessed cortical activity with quantitative EEG with CES intervention for 30 non-clinical volunteers and their findings demonstrated an increase in delta frequency. In contrast, Frankel et al. (1973)^39^ showed no modulation in sleep parameters on EEG with CES treatment for 30 days. Difference in the findings of present study and the study of Frankel et al. (1973)^39^ could be attributed to different study populations (sleep disturbed versus primary insomniacs) recruited for both the studies since pathological process of sleep dysfunction differs in sleep disturbance, which is a sub-clinical entity and insomnia, which is an established clinical condition.

Significant reduction in N1% with CES is another important finding of the present study. As N1 stage serves a transitional role in sleep cycle and is easily interrupted by an external stimulus^9^, reducing N1 facilitates an individual to move to N2 sleep faster. N2 poses more depth of sleep than N1 therefore, CES treatment allows the individual to initiate and maintain a sound sleep. A similar finding was obtained by a previous study^40^, which showed that 24 sessions with CES significantly reduced the length of time that it takes to accomplish the transition from wakefulness to sleep in patients with sleep dysfunction. In contrast to our findings, Wagenseil et al. (2018)^36^ conducted a randomized controlled trial on 40 healthy volunteers and showed that no change in N1% occurred with CES. However, it is to be noticed that Wagenseil et al. (2018)^36^ administered CES device for an hour only before the PSG assessment which could have reflected the acute effects the intervention whereas in the present study 12 weeks of supervised CES sessions were provided to every participant pointing towards the chronic effect of the intervention.

Although, the underlying mechanism of how CES improves sleep is not clear, several theories can be used in an attempt to explain the empirical findings and clinical effectiveness of CES. The brain functions electrochemically and therefore, can be easily modulated by interventions using electric currents^41^. CES intervention stimulates the cortex using low level of AC currents^42^. Several electromagnetic tomography and functional magnetic resonance imaging studies suggests that CES travels to all the cortical and sub-cortical structures including the thalamus^43^. Sleep related problem are thought to be exacerbated by excessive cortical activation^44^. A recent functional magnetic resonance imaging study showed that CES causes cortical deactivation in various regions of the brain after treatment, thus facilitating sleep^45^. CES application has also been shown to modulate neurotransmitters and hormone production via the hypothalamic-pituitary axis.

Increase in the levels of melatonin, norepinephrine and β-endorphin along with reductions in the concentration of cortisol may result in the alleviation of the problems related to sleep^43^. CES treatments also significantly alters EEG activity^46^ such increasing alpha (8-12Hz) relative power and decreasing relative power in the beta (12-30Hz) frequencies. Increased alpha is associated with improved relaxation^47^, whereas decreased beta correlates with reduction in anxiety and stress^48^. Altogether, changes in neurochemicals, deactivation of certain cortical areas, and modulation of brain rhythms may produce relaxation and facilitate sleep function.

The investigators recognize certain strengths and weaknesses in this study. Among the former, is the randomized controlled study design. The nearly even split between the control and treatment cohorts was another strength along with the similar demographics between the two groups. Among the weaknesses, perhaps the main limitation is the sample size. A larger study group might identify more robust findings. Moreover, inclusion of female participants may lead to better generalizability, as this study only recruited male participants for the purpose of convenience. Additionally, sham control group instead of passive control, and blinding the participants would have yielded better and clearer results. Future studies may incorporate aforementioned deficiencies to improve as well as strengthen the results obtained in the present study.

In conclusion, the present study demonstrated that 12-weeks of CES intervention reduced N1% and improved N3%, however no effect was observed in other PSG variables and/or PSQI score. Moreover, the findings of this study also touch on the adherence rate, safety and tolerability of CES for the treatment of sleep disturbance. Findings of the present study leave scope for future research to focus on improving other variables such as SL and SE by identifying the most effective dosage of CES. Moreover, forthcoming investigations should try to build upon the findings of present study so as to strengthen literature pertaining to improvement in sleep through non-pharmacological interventions.

## References

[1] Azad MC, Fraser K, Rumana N, Abdullah AF, Shahana N, Hanly PJ (2015). Sleep disturbances among medical students: a global perspective. J Clin Sleep Med.

[2] Lopresti AL, Hood SD, Drummond PD (2013). A review of lifestyle factors that contribute to important pathways associated with major depression: diet, sleep and exercise. J Affect Disord.

[3] Hamblin JE (2007). Insomnia: an ignored health problem. Prim Care.

[4] Grewal RG, Doghramji K, Attarian HP, Shuman C (2017). Clinical handbook of insomnia.

[5] Daley M, Morin CM, LeBlanc M, Grégoire JP, Savard J (2009). The economic burden of insomnia: direct and indirect costs for individuals with insomnia syndrome, insomnia symptoms, and good sleepers. Sleep.

[6] Hohagen F, Rink K, Käppler C, Schramm E, Riemann D, Weyerer S (1993). Prevalence and treatment of insomnia in general practice. Eur Arch Psychiatry Clin Neurosci.

[7] Foley KA, Sarsour K, Kalsekar A, Walsh JK (2010). Subtypes of sleep disturbance: associations among symptoms, comorbidities, treatment, and medical costs. Behav Sleep Med.

[8] Spira AP, Friedman L, Aulakh JS, Lee T, Sheikh JI, Yesavage JA (2008). Subclinical anxiety symptoms, sleep, and daytime dysfunction in older adults with primary insomnia. J Geriatr Psychiatr Neurol.

[9] Altevogt BM, Colten HR, Institute of Medicine (US) Committee on Sleep Medicine and Research (2006). Sleep disorders and sleep deprivation: an unmet public health problem National Academies Press.

[10] Phillips B, Mannino DM (2007). Do insomnia complaints cause hypertension or cardiovascular disease?. J Clin Sleep Med.

[11] Katz DA, McHorney CA (1998). Clinical correlates of insomnia in patients with chronic illness. Arch Intern Med.

[12] Crönlein T (2016). Insomnia and obesity. Curr Opin Psychiatry.

[13] Knutson KL, Spiegel K, Penev P, Van Cauter E (2007). The metabolic consequences of sleep deprivation. Sleep Med Rev.

[14] Ayas NT, White DP, Al-Delaimy WK, Manson JE, Stampfer MJ, Speizer FE (2003). A prospective study of self-reported sleep duration and incident diabetes in women. Diabetes Care.

[15] Katz DA, McHorney CA (2002). The relationship between insomnia and health-related quality of life in patients with chronic illness. J Fam Pract.

[16] Carter JR, Grimaldi D, Fonkoue IT, Medalie L, Mokhlesi B, Van Cauter E (2018). Assessment of sympathetic neural activity in chronic insomnia: evidence for elevated cardiovascular risk. Sleep.

[17] Pagani M, Pizzinelli P, Pavy-Le Traon A, Ferreri C, Beltrami S, Bareille MP (2009). Hemodynamic, autonomic and baroreflex changes after one night sleep deprivation in healthy volunteers. Auton Neurosci.

[18] Leproult R, Copinschi G, Buxton O, Van Cauter E (1997). Sleep loss results in an elevation of cortisol levels the next evening. Sleep.

[19] Irwin M, McClintick J, Costlow C, Fortner M, White J, Gillin JC (1996). Partial night sleep deprivation reduces natural killer and cellular immune responses in humans. FASEB J.

[20] Killgore WDS (2010). Effects of sleep deprivation on cognition. Prog Brain Res.

[21] Winbush NY, Gross CR, Kreitzer MJ (2007). The effects of mindfulness-based stress reduction on sleep disturbance: a systematic review. Explore.

[22] Brown FC, Buboltz Junior WC, Soper B (2002). Relationship of sleep hygiene awareness, sleep hygiene practices, and sleep quality in university students. Behav Med.

[23] Roehrs T, Roth T (2008). Caffeine: sleep and daytime sleepiness. Sleep Med Rev.

[24] Pack AI, Black JE, Schwartz JR, Matheson JK (2001). Modafinil as adjunct therapy for daytime sleepiness in obstructive sleep apnea. Am J Respir Crit Care Med.

[25] Morin CM, Bootzin RR, Buysse DJ, Edinger JD, Espie CA, Lichstein KL (2006). Psychological and behavioral treatment of insomnia: update of the recent evidence (1998-2004). Sleep.

[26] Kirsch DL, Nichols F (2013). Cranial electrotherapy stimulation for treatment of anxiety, depression, and insomnia. Psychiatr Clin North Am.

[27] Rosenthal SH, Wulfsohn NL (1970). Electrosleep—a clinical trial. Am J Psychiatry.

[28] Empson JA (1973). Does electrosleep induce natural sleep?. Electroencephalogr Clin Neurophysiol.

[29] Rosenthal SH (1972). Electrosleep: a double-blind clinical study. Biol Psychiatry.

[30] Aseem A, Hussain ME (2019). Impact of cranial electrostimulation on sleep: a systematic review. Sleep Vigil.

[31] Buysse DJ, Reynolds III CF, Monk TH, Berman SR, Kupfer DJ (1989). The Pittsburgh sleep quality index: a new instrument for psychiatric practice and research. Psychiatry Res.

[32] Aseem A, Chaudhry N, Hussain ME (2020). Association of P300 event-related potential with sleep in Indian Collegiate Population. Sleep Vigil.

[33] Lichtbroun AS, Raicer MM, Smith RB (2001). The treatment of fibromyalgia with cranial electrotherapy stimulation. J Clin Rheumatol.

[34] Philip P, Demotes-Mainard J, Bourgeois M, Vincent JD (1991). Efficiency of transcranial electrostimulation on anxiety and insomnia symptoms during a washout period in depressed patients a double-blind study. Biol Psychiatry.

[35] Lande RG, Gragnani C (2013). Efficacy of cranial electric stimulation for the treatment of insomnia: a randomized pilot study. Complement Ther Med.

[36] Wagenseil B, Garcia C, Suvorov AV, Fietze I, Penzel T (2018). The effect of cranial electrotherapy stimulation on sleep in healthy women. Physiol Meas.

[37] Hozumi S, Hori H, Okawa M, Hishikawa Y, Sato K (1996). Favorable effect of transcranial electrostimulation on behavior disorders in elderly patients with dementia: a double-blind study. Int J Neurosci.

[38] Kennerly R (2004). QEEG analysis of cranial electrotherapy: a pilot study. J Neurother.

[39] Frankel BL, Buchbinder R, Snyder F (1973). Ineffectiveness of electrosleep in chronic primary insomnia. Arch Gen Psychiatry.

[40] Weiss MF (1973). The treatment of insomnia through the use of electrosleep: an EEG study. J Nerv Ment Dis.

[41] Brunoni AR, Nitsche MA, Bolognini N, Bikson M, Wagner T, Merabet L (2012). Clinical research with transcranial direct current stimulation (tDCS): challenges and future directions. Brain Stimul.

[42] Ferdjallah M, Bostick Junior FX, Barr RE (1996). Potential and current density distributions of cranial electrotherapy stimulation (CES) in a four-concentric-spheres model. IEEE Trans Biomed Eng.

[43] Zaghi S, Acar M, Hultgren B, Boggio PS, Fregni F (2010). Noninvasive brain stimulation with low-intensity electrical currents: putative mechanisms of action for direct and alternating current stimulation. Neuroscientist.

[44] Perlis M, Shaw PJ, Cano G, Espie CA, Kryger MH, Roth T, Dement WC (2011). Principles and Practice of Sleep Medicine.

[45] Devlin JT, Matthews PM, Rushworth MFR (2003). Semantic processing in the left inferior prefrontal cortex: a combined functional magnetic resonance imaging and transcranial magnetic stimulation study. J Cogn Neurosci.

[46] Schroeder MJ, Barr RE (2001). Quantitative analysis of the electroencephalogram during cranial electrotherapy stimulation. Clin Neurophysiol.

[47] Lagopoulos J, Xu J, Rasmussen I, Vik A, Malhi GS, Eliassen CF (2009). Increased theta and alpha EEG activity during nondirective meditation. J Altern Complement Med.

[48] Kučikienė D, Praninskienė R (2018). The impact of music on the bioelectrical oscillations of the brain. Acta Med Litu.

